# Tumor Type Agnostic Therapy Carrying BRAF Mutation: Case Reports and Review of Literature

**DOI:** 10.3390/ph14020159

**Published:** 2021-02-16

**Authors:** Ottavia Bernocchi, Marianna Sirico, Silvia Paola Corona, Carla Strina, Manuela Milani, Maria Rosa Cappelletti, Giuseppina Ferrero, Nicoletta Ziglioli, Valeria Cervoni, Andrea Macchiavelli, Giandomenico Roviello, Daniele Generali

**Affiliations:** 1 Department of Medical, Surgery and Health Sciences, University of Trieste, Piazza Ospitale 1, 34129 Trieste, Italy; silcorona@hotmail.it (S.P.C.); dgenerali@units.it (D.G.); 2 Breast Cancer Unit and Translational Research Unit, Azienda Socio-Sanitaria Territoriale Cremona, Viale Concordia 1, 26100 Cremona, Italy; mmed.sir@gmail.com (M.S.); carlastrina@gmail.com (C.S.); manuelamilani@googlemail.com (M.M.); mariarosacappelletti@gmail.com (M.R.C.); n.ziglioli@asst-cremona.it (N.Z.); valeriacervoni89@gmail.com (V.C.); 3 Department of Anatomical Pathology, Azienda Socio-Sanitaria Territoriale Cremona, Viale Concordia 1, 26100 Cremona, Italy; g.ferrero@asst-cremona.it; 4 Hospital Pharmacy, Azienda Socio-Sanitaria Territoriale Cremona, Viale Concordia 1, 26100 Cremona, Italy; a.machiavelli@asst-cremona.it; 5 Department of Health Sciences, Section of Clinical Pharmacology and Oncology, University of Florence, Viale Pieraccini, 6, 50139 Florence, Italy; giandomenicoroviello@gmail.com

**Keywords:** intrahepatic cholangiocarcinoma (ICC), Bellini duct carcinoma (BDC) BRAF mutation, targeted therapy, next generation sequencing, vemurafenib, cobimetinib, dabrafenib, trametinib

## Abstract

Background: Precision medicine is based on molecular and genotypic patient characterization to define specific target treatment. BRAF mutation is an oncogenic driver, and the Cancer Genome Atlas has identified BRAF mutations in different cancer types. Tumor type agnostic therapy is based on targeting genomic alterations, regardless of tumor origin. In this context, novel therapeutic agents including BRAF and MEK inhibitors based on the molecular landscape in solid tumors have been investigated. Case presentation, Case 1: The first case is chemotherapy-refractory, BRAF V600E mutated intrahepaticcholangiocarcinoma treated with vemurafenib and cobimetinib as third line therapy. In this setting the dual BRAF and MEK inhibition resulted in improved progression-free survival and quality of life; Case 2: The second case shows aBRAF G466A mutated Bellini duct carcinoma (BDC), treated with dabrafenib and trametinib in second line therapy. The disease remained under control for 11 months after the first relapse. Discussion: In the literature there is strong evidence that melanoma, colorectal cancer, non small cell lung cancer and anaplastic thyroid cancer with BRAF mutations are good targets for BRAF/MEK pathway inhibitors. The VE-BASKET and ROAR basket trials explored the efficacy of vemurafenib and the combination of dabrafenib/trametinib, respectively, in BRAF V600 mutation-positive cancers other than melanoma, papillary thyroid cancer, colorectal cancer and non small cell lung cancer. Within the concept of tumor type agnostic therapy, we decided to treat our BRAF-mutated tumors with the association of BRAF and MEK inhibitors. Conclusions: Our results confirm the emerging importance of molecular tumor profiling for the successful management of cancer, and the potential of BRAF-targeted therapy in the treatment of rare solid tumors with poor prognosis and no clinical benefit from systemic therapies with.

## 1. Introduction

Over the past decade, tumor molecular profiling has been widely applied, leading to an individualized approach for patients termed “personalized” or “precision medicine”. This new approach has replaced the standard chemotherapy treatment based on the tumor’s origin, histology and nodal invasion (TNM) [1]. The aim of precision medicine is to define treatment based upon genomic drivers of tumorigenesis, thus identifying the best therapy for patients [2]. Moreover, precision oncology assesses tumor response to a specific treatment and finds drug resistance when it occurs [3]. 

The BRAF mutant is an oncogenic driver, since BRAF inactivation slows down systemic tumor growth and induces cancer cell toxicity [4]. BRAF is a member of the Raf kinase family and plays a critical role in cellular growth, proliferation and differentiation through the MAP-kinase (MAPK) pathway [5]. 

The Cancer Genome Atlas (TCGA) has identified BRAF mutations in many different cancer types: 60% of melanomas, 60% of thyroid cancers, 15% of colorectal cancers, and 5–8% of non-small cell lung cancers [6]. Moreover, mutations in this gene are present in diffuse gliomas, cholangiocarcinoma, hairy cell leukemia, multiple myeloma, Langerhans cell histiocytosis and Erdheim–Chester disease [7]. 

Around 200 BRAF mutant alleles and 30 mutations of BRAF have been identified and characterized and V600E is the most common mutation [8]. BRAF mutations can be classified into three classes: class 1 and 2 are RAS-independent, while class 3 depends on RAS signaling [9]. 

Pembrolizumab and nivolumab for patients with high levels of microsatellite instability and deficient DNA mismatch repair (dMMR), and larotectinib for tropomyosin receptor kinases (TRK), have introduced the concept of tumor type agnostic therapy [10] where genomic alterations could be used to drive tumor therapy, regardless of tumor origin [11]. 

On the basis of this concept, in this report we describe two case studies in which treatment decisions have been driven by next generation sequencing (NGS). The first case study is that of a 59-year-old Caucasian man with BRAF V600E mutated intrahepatic cholangiocarcinoma (ICC). Intrahepatic cholangiocarcinoma is the second most common hepatic cancer and its incidence is increasing worldwide [4]. One of its targetable alterations is a v-Raf murine sarcoma viral oncogene homolog B (BRAF) mutation. While BRAF mutations are rare in pancreato-biliary cancers, they are reported with higher frequency, about 5% of all cases, in ICC [12]. The majority of mutations occur at the V600 position, with V600E observed in 5% of cholangiocarcinoma (CCA) [13]. 

The second case study is that of a 50-year-old, Caucasian woman, with BRAF G466A mutated Bellini duct carcinoma (BDC). BDC is a very rare and aggressive variant of kidney carcinoma, arising from the renal medulla, possibly from the distal collecting ducts of Bellini [14]. BDC is characterized by an aggressive clinical course and an extremely poor prognosis [15]. Less than one-third of patients survive more than 2 years after diagnosis, and in most reported cases metastatic disease is present ab initio. Radical nephrectomy is generally performed, followed by medical treatments such as immunotherapy, targeted therapies or chemotherapy [16]. Given the rarity of this cancer, it has been difficult to conduct large-scale clinical trials and a specific standard treatment has not yet been established.

These clinical cases may represent a proof of concept of how identifying and targeting potential molecular drivers, such as BRAF mutations, independently of tissue origin, could provide represent therapeutic opportunities, especially for rare cancers.

## 2. Case Presentation

### 2.1. Case 1

A 59-year-old Caucasian male patient, with metabolic syndrome and type 2 diabetes, was admitted to the emergency department in January 2017 with severe hip pain, nausea and increasing discomfort. Abdominal ultrasound and computerized axial tomography showed the presence of a 6 × 9 cm hepatic nodule in the VI segment. In February 2017 the patient underwent VI hepatic segment resection and cholecystectomy. Immunohistochemistry revealed moderately differentiated CCA, positive for CK7, CK19 and CA19, and negative for CK20. The patient was diagnosed with cT2aN0M0 disease. Indication to standard follow-up was given due to the absence of resection margins and lymph node involvement. However, in September 2017, after seven months of follow-up, positron emission tomography (PET) showed hyperfixation of the ischio-pubic branch and sacro-iliac articulation. The subsequent ischio-pubic biopsy revealed cholangiocarcinoma metastasis. At this point, first line chemotherapy with cisplatin (25 mg/m^2^) and gemcitabine (1000 mg/m^2^) on day 1 and 8 every 3 weeks was administered for 6 cycles. Furthermore, palliative radiotherapy was performed on the bone metastasis (20 Gy in fraction 5). After 6 cycles of chemotherapy, CT scans showed hepatic, lung, lymphonodal and bone disease progression. In June 2018, after multidisciplinary discussion, the patient started a second line chemotherapy with oxaliplatin (130 mg/m^2^) on day 1 and capecitabine (1000 mg/m^2^) orally BID for 14 days every 3 weeks, without clinical and radiological response.

**Figure 1:**
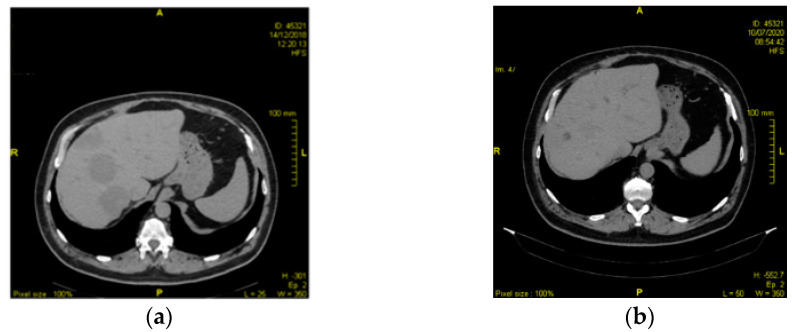
(**a**) CT scan, March 2019; (**b**) CT scan, September 2020.

In light of tumor resistance to second line chemotherapy, primary tumor next generation sequencing was performed in January 2019, which showed the presence of a BRAF V600E mutation. Given this result, it was decided to start off-label use of vemurafenib (960 mg/day) and cobimetinib (60 mg/day). During the course of this therapy, he remained almost completely asymptomatic, with the exception of some episodes of grade 2 nausea and self-limiting skin rash, which occurred during the first month. Biochemical evaluations showed an increase in alkaline phosphatase and blood creatine phosphokinase during the first and second month, respectively. Six months after beginning targeted therapy, restaging CT scans reported a treatment response, with an additional reduction of pulmonary nodules and hepatic lesions. Furthermore, the bone lesions appeared sclerotic due to treatment response. His last CT scan was performed in September 2020 and confirmed stable disease (Figure 1). To date, the patient is still on treatment.

In addition, EORTC QLQ-C30 (a standardized quality of life questionnaire) was conducted both at the beginning of treatment and after the latest radiological evaluation, revealing a progressive improvement in physical functioning (essential abilities for maintaining independence) and role functioning (abilities for work/leisure). Moreover, the patient has gradually reduced opioid intake until complete suspension.

### 2.2. Case 2

In January 2015, a 50-year-old, Caucasian woman, without comorbidities, was admitted to our hospital with a painless gross hematuria lasting 3 months. Physical examination revealed a percussion pain over the left kidney region and no extrarenal manifestations were found at clinical and laboratory examinations. A kidney tumor was suspected on the basis of the urinary system ultrasonography and the finding was subsequently confirmed with a total body CT scan. The imaging revealed a 3 × 3.5 cm heterogeneous enhancing mass, in the middle pole of the left kidney, which was further compounded with multiple metastases in the retroperitoneal lymph nodes, ovaries and a 12 mm temporal lobe brain lesion without perilesional edema. The patient subsequently underwent laparoscopic nephroureterectomy.

The final pathological report showed Bellini duct carcinoma (BDC) of the left kidney, Fuhrman Nuclear Grade IV. The histological diagnosis of BDC was confirmed by positive immunohistochemical staining with UEA-1 and EMA, and negative staining with Leu-M1 [17]. According to the American Joint Committee on Cancer (AJCC) 2015 classification and TNM classification, the patient was diagnosed with a high-grade, stage 4 disease. 

After multidisciplinary discussion, in February 2015, the patient started first line chemotherapy with bevacizumab (15 mg/kg) and gemcitabine (1250 mg/m^2^) on days 1 and 8 and platinum salt (cisplatin 80 mg/m^2^ or carboplatin AUC 5 mg/ml/min) every 3 weeks. After 3 cycles, she obtained a clinical response and a CT scan showed partial radiological response. At this stage, one of the ovarian metastasis was resected in order to create a patient-derived xenograft (PDX). At the end of the 5th cycle, she developed febrile neutropenia (neu < 500/mm^3^). In light of the hematological toxicity, the chemotherapy was stopped while maintaining bevacizumab every 3 weeks. Maintenance therapy was well tolerated. Furthermore, stereotaxic radiotherapy was performed on the brain metastasis (24 Gray in 2 fractions).

After 6 cycles of maintenance bevacizumab (December 2015), retroperitoneal lymph node disease progressed. Using the PDX mouse-derived model, we were able to perform a mutational analysis. 

**Figure 2:**
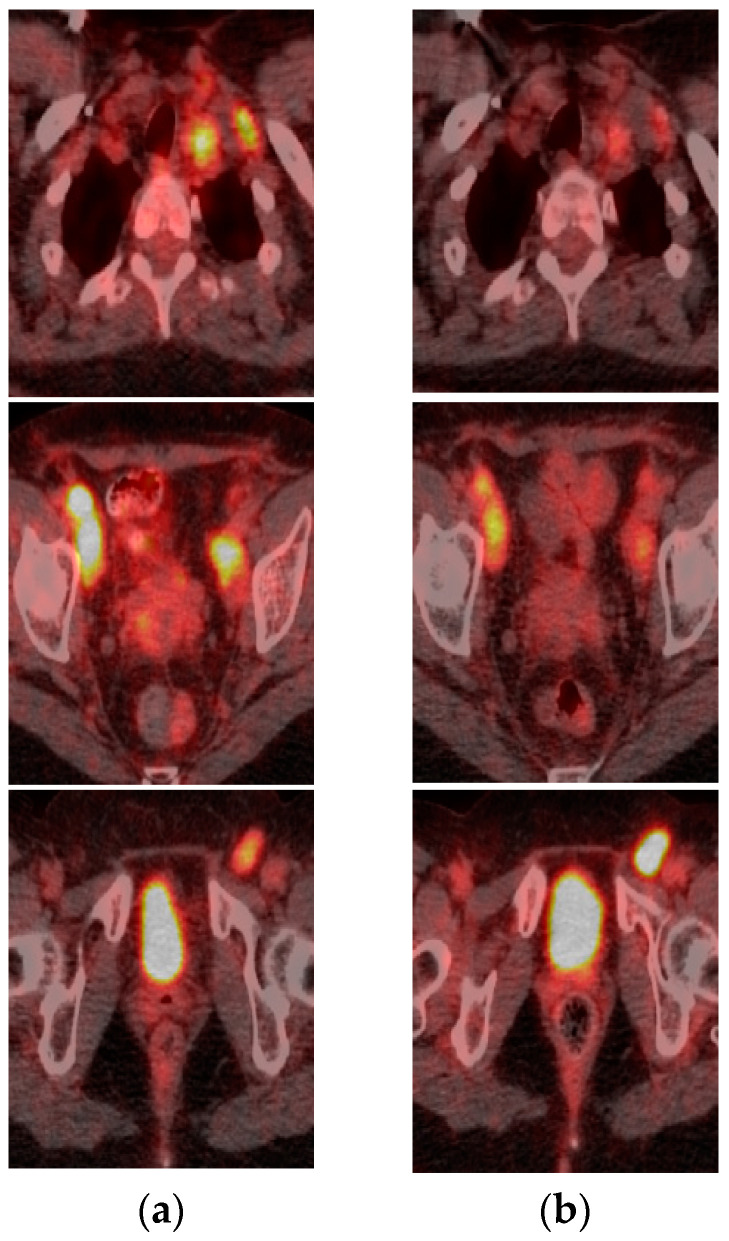
FDG-PET after 4 weeks of treatment with dabrafenib and trametinib showed decreased FDG avidity. (**a**) FDG-PET performed in January; (**b**) FDG-PET performed in February 2016.

Genomic sequencing of the ovarian metastasis revealed a BRAF G466A mutation. Hence, a therapeutic combination of trametinib and dabrafenib was started. After one month of therapy, the patient developed pyrexia and rash treated with common medications; however, the 18F-FDG-PET/CT performed at the beginning of treatment and repeated after 4 weeks showed decreased FDG avidity in the ovary and lymph node metastases (Figure 2).

From January 2016 to December 2016 the patient showed stable disease. However, in January 2017, she was admitted to the emergency department complaining of headaches, nausea and dizziness; her performance status worsened to 3, requiring the patient to be hospitalized. Brain CT scans revealed multiple metastases in the frontal, parietal and temporal lobe bilaterally, further confirmed by MRI. After one week, the patient passed away due to widespread brain metastasis. The disease was under control for 11 months after the first relapse.

## 3. Discussion

In 2011, the Food and Drug Administration approved the BRAF inhibitor vemurafenib for the treatment of BRAF V600E mutant metastatic melanoma [18]. Dabrafenib, another BRAF inhibitor, obtained FDA approval in 2013 [19]. In the coBRIM trial, Larkin J et al. demonstrated that the combination of vemurafenib with the MEK inhibitor cobimetinib resulted in significant progression-free survival (PFS) improvement among patients with BRAF V600E mutated metastatic melanoma in comparison to the combination of vemurafenib with placebo [20]. More recent studies showed that combining BRAF and MEK inhibitors determines a PFS improvement from a median of 7 months to 12 months in this setting [21]. Furthermore, the combination of MEK and BRAF inhibitors can decrease the onset of resistance to treatment and side effects that arise during BRAF inhibitor monotherapy [22]. This combination reduces the incidence of skin toxicity, including cutaneous squamous carcinoma associated with BRAF inhibition [23]. In mutated cells, B-raf protein is found in its hyperactive form. Combined BRAF inhibitors block the Ras-Raf-MEK-MAPK kinase pathway, but, as it happens with monotherapy, pharmacological resistance may develop after 6–7 months of treatment. To overcome this resistance, BRAF inhibitors are used in combination with MEK inhibitors which mediate the blockade of MAPK kinase (MEK), downstream of BRAF in the MAPK pathway [24]. Beside melanoma, thyroid cancers [25] and colon cancers [26], the prevalence of a BRAF V600E mutation in other cancers is less than 5% [27]. The relatively low frequency of this mutation accounts for the lack of randomized controlled studies looking at the use of specific anti-BRAF targeted therapies in other cancers. This difficulty is even true in the case of very rare tumors such as CCA and BDC, so that tumor-specific studies are almost impossible to run. On the other hand, the lack of therapeutic options for these rare diseases provides a strong rationale for targeting putative driver mutations.

To address such paradoxes, basket trials have come to the rescue, putting together tumors with different histology, on the basis of their molecular landscapes and, more specifically, the presence of a BRAF V600E mutation [28]. Whilst this approach still has limitations, such as the small number of patients enrolled per tumor type, and therefore there is low statistical power in the studies, some important indications can be drawn from them. Hyman et al. report results from a basket trial including a total of 122 patients with BRAF V600E mutation-positive cancers [29]. The primary end point of the study was the response rate; secondary end points were progression-free survival and overall survival. As the response varied across tumor types, the authors concluded that BRAF V600E mutation is certainly targetable in many, but not all, cancers, and that perhaps the tumor site in which they develop are important in determining the degree of response to a specific targeted therapy. The study had 6 tumor cohorts plus an “all other cancers” cohort. Clinical activity, including some complete responses, was observed in NSCLC, Langerhans cell histiocytosis, anaplastic thyroid cancer, ovarian cancer and cholangiocarcinoma. Colon cancer patients did not show any response to vemurafenib single-agent therapy and even when the anti-BRAF therapy was combined with cetuximab the results were not encouraging. These patients were however heavily pretreated, having had 1 to 6 previous lines of therapy [29]. The contribution of mutated BRAF seems to change amongst tumor types, as suggested by the very heterogeneous results in terms of clinical benefit of BRAF inhibition in different cancers. For instance, this mutation alone may not sufficient to drive tumor progression in certain tumor types, such as colon cancer [30]. 

Thus, the question of whether all BRAF-mutated cancers are amenable to specific targeted therapy is still open. Perhaps, extremely rare tumors and orphan cancers, where proven effective treatment strategies are lacking, could benefit by even modest therapeutic activity. Cholangiocarcinoma is one of the most aggressive biliary tract malignancies [31]. After first-line therapy, no standard second-line treatment has been established yet and the outcome remains rather poor, with a 5-year overall survival rate of around 2% due to metastatic disease [32]. Data from the literature show that nearly 35% of CCA harbor potentially targetable genomic alterations and the use of molecular profiling has led to the discovery of potential disease drivers [33]. Lowery MA et al. showed that IDH1 (25%), TP53 (24%), ARID1A (21%), BAP1 (15%), KRAS (13%), PBRM1 (12%), SMAD (9%), ATM (8%), BRAF (<5%) and MSI-H (0.5%) are the most commonly mutated genes in CCA [13]. Biliary tract cancer reveals molecular heterogeneity and there is a crucial need to identify a subset of patients who can benefit from targeted therapy to be used after progression on first-line treatment gemcitabine plus cisplatin [34]. The ClarIDHy study showed that ivosidenub significantly improved PFS, with a trend towards favorable overall survival, in comparison to placebo in patients with advanced cholangiocarcinoma carrying an IDH1 mutation [35]. At ASCO GI 2019, Park et al. presented encouraging preliminary results on the efficacy of erdafitinib in FGFR-mutated CCA patients as a second-line therapy (NCT 02699606). Andersen et al. reported that BRAF V600E occurs with the highest frequency in ICC (1.5%) and is associated with poor prognosis [36]. Focusing on biliary tract cancer, preliminary data from the ROAR basket trial demonstrated promising activity of dabrafenib plus trametinib with a favorable safety profile in patients with BRAF V600E (ROAR trial; NCT 02034110).

Based on all these results, we decided to administer a combination of MEK and BRAF inhibitors in a patient with BRAF mutant, chemotherapy-refractory ICC and obtained results in line with the few cases reported in literature. Lavingia et al. described two cases reaching 9 and 12 months PFS when treated with dabrafenib and trametinib [37]. Bunyatov et al. showed the longest remission with the same combination: a full response after 7 months of treatment with complete remission lasting 28 months [38]. In the second case, the PDX model predicted clinical response to dabrafenib and trametinib. BDC is a rare subtype of renal cell carcinoma with an extremely poor prognosis. Given the rarity of BDC and therefore the scarcity of specific clinical trials, the best treatment for this tumor remains uncertain. The only combination of cytotoxic agents which shows effectiveness is gemcitabine plus platinum [39]. Oudard et al. showed a 26% objective response in BDC with the gemcitabine plus platinum salt regimen used as a first-line treatment [40]. Furthermore, recent data also suggest a role for anti-angiogenic therapy in this setting. Péchuchet et al. demonstrated that addition of bevacizumab to platinum-based chemotherapy in previously untreated BDC patients resulted in longer PFS and OS, with a manageable safety profile [41]. In his BDC case report, El Mehdi Tazi et al. demonstrated a therapeutic response to sunitinib [42] and Ansari J et al. showed similar results with sorafenib [43]. Unfortunately, clinical trials failed to show activity of any targeted drugs [44]. Sharing common morphological and biological characteristics with urothelial carcinomas, to date, chemotherapy remains the standard treatment for patients with BDC. Nevertheless, after progression with first-line chemotherapy, the second-line approach still presents a challenge, and patient outcome remains poor. For this reason and on the basis of the genomic sequencing results, with the presence of a BRAF G466A mutation, we decided to use the combination of anti-BRAF and anti-MEK treatment. G466A belongs to Class 3 BRAF mutations depending on RAS signaling: thus, blocking RAS signaling would appear to be a potential therapeutic strategy for class 3 BRAF-mutant tumors, but to date there are no specific inhibitors available [45].

## 4. Conclusions

In the last few years, there have been increasing efforts to understand the molecular biology of several rare tumors with limited treatment options. Genome analysis could, in these cases, help find specific genomic alterations which can be targeted. Precision medicine allows a personalized approach based on biomarker individualization [46].

The approval of the first-in-class tumor agnostic therapies shows that identification of biomarkers independent from tumor origin could offer new treatment options with drugs already on the market [47].

In the literature, there is strong evidence that different tumors with BRAF mutations are good targets for BRAF/MEK pathway inhibitors and our work adds to this evidence.

This report highlights the importance of molecular profiling for the management of solid tumors, and more specifically, orphan tumors. In the era of precision medicine, patients with otherwise very grim expectations can benefit from such approaches while we wait for more prospective studies to provide evidence. 

## Data Availability

Data is available from the authors upon request.
